# Fingering instabilities in tissue invasion: an active fluid model

**DOI:** 10.1098/rsos.181579

**Published:** 2018-12-19

**Authors:** Michał J. Bogdan, Thierry Savin

**Affiliations:** Department of Engineering, University of Cambridge, Cambridge, UK

**Keywords:** active fluids, tissue invasion, metastasis, fingering instabilities

## Abstract

Metastatic tumours often invade healthy neighbouring tissues by forming multicellular finger-like protrusions emerging from the cancer mass. To understand the mechanical context behind this phenomenon, we here develop a minimalist fluid model of a self-propelled, growing biological tissue. The theory involves only four mechanical parameters and remains analytically trackable in various settings. As an application of the model, we study the evolution of a two-dimensional circular droplet made of our active and expanding fluid, and embedded in a passive non-growing tissue. This system could be used to model the evolution of a carcinoma in an epithelial layer. We find that our description can explain the propensity of tumour tissues to fingering instabilities, as conditioned by the magnitude of active traction and the growth kinetics. We are also able to derive predictions for the tumour size at the onset of metastasis, and for the number of subsequent invasive fingers. Our active fluid model may help describe a wider range of biological processes, including wound healing and developmental patterning.

## 1. Introduction

Spreading tumours often do not maintain a straight front while expanding. They instead display an interface patterned with multicellular protrusions, which are commonly referred to as fingers [1,2], invading the surrounding tissue [1–6]. Their formation generally initiates cancer metastasis [2–4,7,8], which is responsible for the vast majority of cancer-related deaths [9]. Similar structures form during wound healing, where fingers accompany re-epithelization [10,11]. In the case of glioblastoma brain tumours, these fingers usually consist of disconnected, diffusing cells [1,12]. But in carcinomas and epithelial wound healing, they tend to remain condensed, with a well-defined boundary [5,10].

What causes the formation of such fingers? Studying cancer has traditionally focused on a large number of biological (especially genetic and biochemical) cues [13]. Yet, these essentially operate by collectively affecting a smaller number of physical properties of the tissues and environments involved [1]. How these physical alterations can, in turn, lead to fingering has been investigated by several models. Various causal mechanisms, differing in their assumptions on the mechanical properties adopted for the tissues, have been proposed [14–20]. For example, reaction–diffusion models of nutrient-limited growth have been used [1,21] because the accessibility of diffusing chemicals is necessary for tumour growth. But, other models have treated the fingers’ emergence as a consequence of a mechanical instability [14,15,17,22]. Support for the latter approach was provided by experiments conducted to prevent biochemical signalling, but in which fingering occurred nevertheless [23]. Mechanical processes proposed by these models include: fracturing of an elastic surrounding medium (extracellular matrix, healthy tissue) upon pressure from a growing solid inclusion (tumour), leading to subsequent infiltration of the cracks by malignant cells [22]; branching instabilities caused by the tumour’s confinement pressure, analogous to the corona splash of a drop impacting a solid surface [24]; mechanical frustrations between an outer, proliferating ring of a growing tumour and its necrotic core [15]; buckling due to swelling of a spatially restricted gel-like tissue [17]; instability resulting from the interplay between spatially non-uniform cell division/death rates and shear in viscous tissues [14]; or a pulling mechanism by a subpopulation of leader cells at the tumour’s edges [10,25].

Many of the above mechanisms have been successfully described using continuum, analytically solvable models [14–17], thus providing deep insights into the physical context involved in tissue fingering. However, these continuum models of tumours have, in most cases, omitted one fundamental component of live tissues: cells actively apply forces (via the conversion of chemical energy into mechanical energy) to their surroundings [10,26–30]. Yet, the onset of fingering at tissue boundaries is often correlated with the dense presence of these so-called active forces and the occurrence of the resulting self-propelled motion [10,29]. In discrete models, the central role of these forces in triggering tissue fingering has been verified by simulations within several different frameworks [18,25]. But, to the best of our knowledge, only few continuum models of fingering have studied the consequences of self-propelling forces: the effect of an active rim at the boundary of the tissue has been studied by Mark *et al.* [31] and by Nagilla *et al.* [32], while the role of active forces in the tissue bulk has been discussed in wound healing models by Zimmermann *et al.* [33] and by Nesbitt *et al.* [34]. However, the analyses in the two latter works are limited to a non-dividing, rectangular tissue, either in a static state or somehow pushed on one side by a rigid barrier.

We here investigate the role of tissue bulk activity for the emergence of fingers in more general situations. We first construct a continuum mechanical model of an active and growing tissue, supported by experimental evidence, and that is analytically solvable and that involves only four physical parameters: friction, activity, growth and surface tension. We next investigate the role of activity in promoting fingering. Provided with experimentally derived estimates of the physical parameters, our model notably produces realistic predictions for the number and evolution of the fingers.

## 2. Model

### 2.1. Assumptions and equations

In our model of an active and growing tissue, the evolutions of the pressure *p*(**r**, *t*) and velocity **v**(**r**, *t*) fields at a position **r** and time *t* are governed by the following force balance and mass conservation equations,

2.1*a* $$\nabla p=-\beta v+\alpha \frac{v}{\left|v\right|}$$

and

2.1*b* ∇⋅v=k,

respectively, with ∇ the nabla operator, and where *α* and *β* are positive parameters, specifying the strength of the interaction between the tissue and a substrate: *α* describes the magnitude of the active traction, while *β* describes the magnitude of the effective passive friction (proportional to the tissue viscosity). In equation (2.1*b*), *k* is the net rate of growth (we are here interested in regimes in which it is also positive) of an incompressible tissue, undergoing cell division (or individual cell growth). The *α*-term in equation (2.1*a*), which accounts for the tissue activity, is discussed in detail in §2.2. The evaluation of the various parameters is examined in §2.3.

Ignoring the *α*-term, equation (2.1*a*) reduces to Darcy’s Law ∇p=−βv (originally used to describe viscous flows in porous materials and Hele–Shaw apparatus [35]), which has been widely employed to model the passive behaviour of tissues [1,36–38]. Darcy’s Law notably assumes a *viscous* and *quasi-two-dimensional* dynamics for the deformations of a tissue layer, by considering that the effects of friction against a substrate are much stronger than those of viscous shear within the plane of the layer.

Using two-dimensional models is experimentally justified by the large prevalence of *in vitro* tissue culture monolayers, and also because many *in vivo* soft tissues, including epithelium in which carcinomas develop, tend to spontaneously form quasi-two-dimensional monolayers [10,29,39]. Consequently, two-dimensional descriptions are often employed in tissue mechanics models [14,17,25].

The mechanical properties of live tissues at short timescales, up to the order of minutes, are generally dominated by an elastic constitutive behaviour. At longer timescales, however, a viscous description is better suited [30]. The crossover between the two regimes is probably related to the turnover rates of intercellular adherent junctions [40,41]. Hence, epithelial tissues become fluidized by a reduction in the number of adherent junctions, and a concomitant increase in the magnitude of active traction when becoming malignant. This well-known ‘melting’ process is often referred to as the ‘epithelial to mesenchymal transition’ [3,5,42]. Moreover, cell division and apoptosis are also known to further contribute to the fluidization of tissues [43]. As we here model the behaviour of the tissue at timescales on which it experiences substantial growth (that is, on the order of several hours at least [4]), the viscous constitutive behaviour implied by Darcy’s Law is justified. Many existing continuum models of epithelial tissues indeed make the same assumption [14,30,38,44]. Note that, by writing equations (2.1), we further assume that inertial terms are negligible on these timescales.

### 2.2. Tissue activity

The second term on the right-hand side of equation (2.1*a*) accounts for cells actively propelling themselves by exerting traction against the substrate. It will subsequently be referred to as the active term, and *α* specifies its strength.

**Figure 1.**
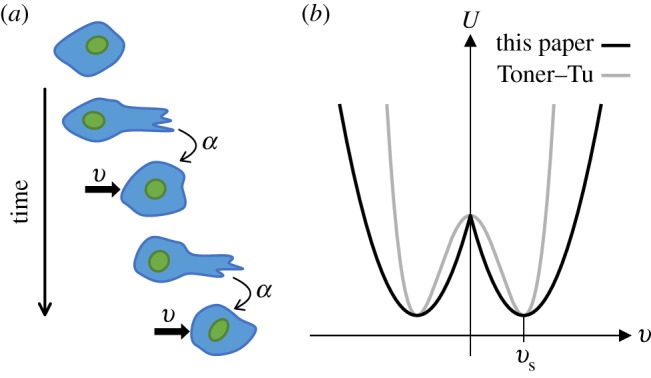
Assumptions of the active traction model. Panel (*a*) is a schematic illustrating how the active traction of a mesenchymal tumour cell acts in the direction of its migration velocity; filopodia and/or lamellipodia are protruding at the leading edge of the cell, which translocates in the same direction. Panel (*b*) compares (in one dimension) the Landau-type ‘velocity potential’ [46], *U*(**v**) such that the volumic traction force **F** = −∂_**v**_*U*, of our description with the Toner–Tu model; both potentials select an intrinsic velocity $v_{s}$ (see text), but our model introduces a discontinuity at **v** = 0 which has no effect on our results.

We consider here that the direction of the net local active force acting on the tissue layer from the substrate is aligned with the direction of the local flow velocity. This assumption was made in previous studies modelling active tissues [18,45]. It is a consequence of cells attempting to maintain their direction of motility, as illustrated in figure 1*a*, and also manifested by the persistent Brownian motion of individual cells *in vitro* [47]. On a subcellular level, it probably results from the friction destabilizing lamellipodia [11,48] that are not aligned with the cell’s velocity [18]. It has further been shown to be consistent with experimental data [18,49,50], although this directionality is not universal [51] and other models have been proposed where the direction of the active force is treated as an independent internal variable, coupled to stress and velocity fields [44,52].

Equation (2.1*a*) also assumes that the active traction does not depend on the magnitude of the velocity. This assumption has been made in several numerical models of motile cells [18,53,54], and enables a distinct analysis of the role played by activity.

In the classic theory derived by Toner & Tu [55,56], often used to model active fluids [33,34,57], the net force per unit volume acting on the active fluid from the substrate, in a spatially uniform flow, follows **F**_T_(**v**) = *α*_T_**v** − *β*_T_|**v**|^2^**v** (ignoring here additional inertial and gradient terms; with *α*_T_ and *β*_T_ positive parameters). In comparison, our model, equation (2.1*a*), gives this force the expression **F**(**v**) = *α***v**/|**v**| − *β***v**. In both cases, the fluid has a ‘preferred’ spontaneous magnitude of velocity |**v**_s_| = *α*/*β* (= (*α*_T_/*β*_T_) ^1/2^ in the Toner–Tu model), which it would select when moving in unbounded space without being driven by an external pressure gradient or growth. For magnitudes of velocity lower than |**v**_s_|, the fluid would be driven to move faster by the *α*-term, while above it, it would be slowed down by the friction (the *β*-term). This fluid’s constitutive behaviour may be described in terms of a Landau-type ‘velocity potential’ [46], shown in figure 1*b*. Although our model has a discontinuity in the direction of **F**(**v**) at **v** = 0 which does not exist in **F**_T_(**v**), we have verified that this singularity does not significantly affect our subsequent results.

The Toner–Tu model, however, assumes that the friction force grows as |**v**|^3^ and that the active force varies linearly with |**v**|. Both assumptions are unrealistic when describing biological tissues. Our model, however, retains the physical interpretation of *β* as the friction coefficient of Darcy’s Law, and of *α* as the magnitude of the active force of the tissue against the substrate (per unit volume). Our approach also enables a direct comparison with classical results for viscous fingering [58], readily obtained from our model by taking the limit *α* → 0.

### 2.3. Estimation of parameters

Based on *in vivo* microscopy observations [4] of the time necessary for doubling a carcinoma’s size, which is on the order of a few hours, we estimate that the growth rate *k* is about 10^−4^ s^−1^.

The passive friction *β* can be estimated based on *in vitro* force measurements of epithelial tissues against substrates [29,30,59] (admittedly, inferences about *in vivo* systems from these *in vitro* experiments are arguable). Following Pompe *et al.* [59], we assume that friction with the substrate is primarily the consequence of cell-substrate ligands, numbering 200–300 per cell, each of which exerts a force of about 10^−12^ N. We thus estimate the total friction force per cellular volume to be about 10^6^ N m^−3^ for a 10^−5^ m cell size. From *in vivo* microscopy of micrometastasis growth [4], the typical velocity *v* of the cells falls within 10^−10^ to 10^−9^ m s^−1^. Hence, dividing the volumic friction force by this velocity provides an estimate *β* ∼ 10^15^–10^16^ Pa s m^−2^.

There is no lower limit on *α*, as epithelial cells may not exert any active force against the substrate. The upper limit can be estimated on the basis of force tracking microscopy applied to spreading epithelial monolayers *in vitro* [29,30]. It is observed that traction forces are actively exerted through the monolayers and peak at their edges, giving rise to a gradient of the stress tensor’s diagonal terms, which is up to 10^7^ Pa m^−1^ in the study by Trepat *et al.* [29], and 10^8^ Pa m^−1^ in the work of Blanch-Mercader *et al.* [30]. Balancing *α* with this typical stress gradient, one can place an upper estimate on *α* at approximately 10^8^ Pa m^−1^. In agreement, the traction exerted by single fibroblasts has been reported as up to 10^−7^–10^−5^ N per cell [60], which would correspond to *α* ∼ 10^8^–10^10^ Pa m^−1^ when dividing by the cell’s volume.

The effective surface tension of the tissue *γ* will also play a role in our further considerations. Its magnitude depends on the strength of intercellular adhesion and behaviour of cortical actin networks [61,62]. The surface tension was evaluated indirectly by Foty *et al.* [63] by measuring the energetic penalty of compression of embryonic multicellular spheroids, revealing values on the order of 3–9 mPa m. We thus presume that *γ* ∼ 10^−3^–10^−2^ Pa m is a realistic range for our system. A summary of the estimates for the physical parameters is presented in table 1.

**Table 1.** Estimates of the physical parameters.

parameter	symbol	unit	value
growth rate	*k*	s^−1^	10^−4^
passive friction	*β*	Pa s m^−2^	10^15^ to 10^16^
active traction	*α*	Pa m^−1^	0 to 10^10^
surface tension	*γ*	Pa m	10^−3^ to 10^−2^

### 2.4. Model system

**Figure 2.**
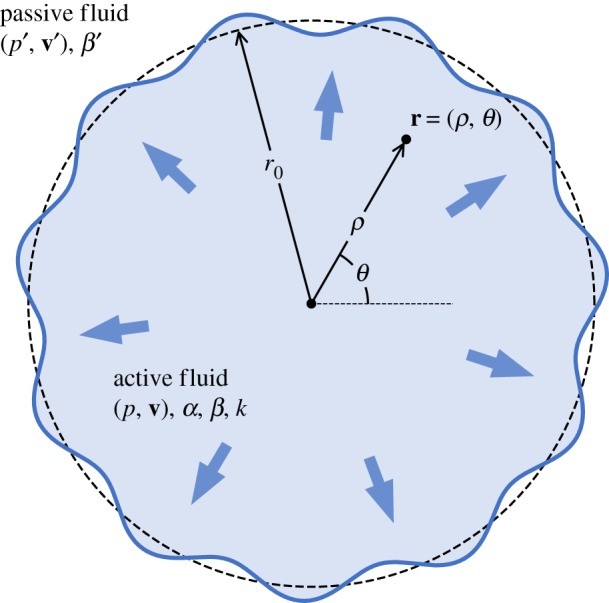
Model system of a tumour growing in an external tissue: a two-dimensional circular droplet with radius *r*_0_ and made of an active fluid described by equations (2.1), is expanding in a passive fluid modelled by equations (2.2). The interface undergoes periodic perturbations whose linear stability is investigated in §3.

The tumour is modelled as an initially circular, two-dimensional droplet of a growing active fluid described by equations (2.1), with an unperturbed, time-dependent radius *r*_0_(*t*) (figure 2). The surrounding healthy tissue is modelled as a passive, non-dividing fluid, whose pressure *p*′(**r**, *t*) and velocity **v**′(**r**, *t*) fields follow the equations:

2.2*a* $$\nabla p^{\prime }=-\beta^{\prime }v^{\prime }$$

and

2.2*b* $$\nabla \cdot v^{\prime }=0,$$

where *β*′ is the friction parameter (analogous to *β* in the active fluid). In writing equation (2.2*b*), we effectively assume that growth in the passive fluid can be neglected on the timescale of metastasis initiation.

We assume that the activity *α* and the growth rate *k* are constant (independent of **r** and *t*) through the active tissue. Constant magnitude of the active force has been assumed in models of active matter before [64,65]. While not correct in all situations [30,66], it is a convenient assumption to evaluate the influence of its magnitude in fingering. Extensions to non-uniform and time-dependent behaviours of these parameters are readily possible, and we investigate a case of evolving growth rate in §3.4 (see also appendix B.2). Similarly, we assume that *β* and *β*′ are uniform within their respective regions.

We study the system in polar coordinates **r** = (*ρ, θ*) and write vector fields’ components in this system with appropriate subscripts, such as $v=(v_{\rho },v_{\theta })$. The perturbed interface between the active and passive fluids, described by the line *r*(*t*, *θ*), must satisfy two boundary conditions. First, the continuity of the radial components of velocities is expressed as follows:

2.3 $$v_{\rho }{|}_{\rho =r}=v_{\rho }^{\prime }{|}_{\rho =r}=\partial_{t}r.$$

Second, the pressure difference across the interface separating the two tissues must equal the Laplace pressure,

2.4 $$p{|}_{\rho =r}-p^{\prime }{|}_{\rho =r}=-\gamma \frac{r^{2}+2{\left(\partial_{\theta }r\right)}^{2}-r\partial_{\theta \theta }^{2}r}{{\left[r^{2}+{\left(\partial_{\theta }r\right)}^{2}\right]}^{3/2}},$$

with *γ* the surface tension, and the fraction being the expression of the local interfacial curvature in polar coordinates [67]. Note that our description does not account for an interfacial bending force [31,68]. We indeed assume that the corresponding energetic cost, which involves a higher order dependency in interfacial curvature when compared with surface tension [31], is negligible at the length scales considered for the linear stability analysis presented in §3.

Unless stated otherwise, we use the dimensionless variables defined as follows. Distances are re-scaled by the characteristic length $\ell ={\left(\frac{2\gamma }{\beta k}\right)}^{1/3}$, which can be interpreted as a capillary length at which growth balances interfacial tension (on the order of 10 µm, based on the estimates of table 1). Times are re-scaled by *k*^−1^, and we further define *ϕ* = *β*′/*β* the relative viscosity of the displaced tissue compared to the active growing droplet. We introduce a reference activity $\alpha^{*}=\beta \ell k∼10^{7}\;\text{Pa}\;{\text{m}}^{-1}$ to make the active traction *α* dimensionless, $\alpha /\alpha^{*}\to \alpha$. Pressures and velocities are made dimensionless by $p^{*}=\beta \ell^{2}k∼10^{2}\;\text{Pa}$ and $v^{*}=\ell k∼10^{-9}\;\text{m}\;{\text{s}}^{-1}$, respectively. We will use the same letters for the dimensionless versions of the variables as for their dimensional counterparts.

The droplet of the active tissue grows due to a positive *k*, as required by equation (2.1*b*), and the passive fluid is displaced by it. As long as the interface between the two tissues remains circular (with the unperturbed radius *r*_0_), hydrodynamic fields in both regions remain symmetric under rotations and are given by

2.5*a* $$v_{0}=\left(\frac{\rho }{2},0\right),\;p_{0}=\alpha (\rho -r_{0})-\frac{1}{4}(\rho^{2}-r_{0}^{2})+p{|}_{\rho =r_{0}}$$

and

2.5*b* $$v_{0}^{\prime }=\left(\frac{r_{0}^{2}}{2\rho },0\right),\;p_{0}^{\prime }=-\frac{\phi r_{0}^{2}}{2}\ln\left(\frac{\rho }{r_{0}}\right)-\frac{1}{2r_{0}}+p{|}_{\rho =r_{0}},$$

as obtained by solving equations (2.1)–(2.4) and using the dimensionless quantities defined above.

## 3. Results and discussion

### 3.1. Linear stability analysis

We investigate under which conditions the active, circular droplet of radius *r*_0_ would start to form finger-like protrusions at its edge, while undergoing uniform growth. To do this, we perform a linear stability analysis around the circular solution given in equations (2.5), by investigating infinitesimal interfacial perturbations of the form *r* = *r*_0_ + *δr*, with

3.1 $$\delta r\propto f_{n}(t)\;{\text{e}}^{in\theta },$$

for an integer *n* corresponding to the mode of the periodic perturbations, and where *f*_*n*_(*t*) is a function describing its time evolution (with lim _*t*→0_ *f*_*n*_(*t*) = 1 for all *n*). An analogous ansatz of periodicity in *θ* is made for the perturbations of hydrodynamic fields in both fluids, (*δp*, *δp*′, *δ***v**, *δ***v**′) ∝ *f*_*n*_(*t*) e^i*nθ*^, around the solution given by equations (2.5). Note that our stability analysis concerns small perturbations around solutions that are themselves time-dependent. Applying the evolution equations, equations (2.1)–(2.4), to these perturbed fields provides an expression for the *n*-mode’s rate of growth defined by

3.2 $$\sigma_{n}(r_{0})=\lim_{t\to 0}\frac{\partial_{t}f_{n}(t)}{f_{n}(t)}.$$

Positive values of σ_*n*_(*r*_0_) correspond to unstable, growing modes *n*, which may become the basis for the formation of fingers. We derive in appendix A the following expression for σ_*n*_(*r*_0_):

3.3 $$\sigma_{n}(r_{0})=\frac{1}{2}\frac{\left(\phi -1)(n-1)-n(n^{2}-1)/r_{0}^{3}+\Lambda_{n}(\frac{2\alpha }{r_{0}}\right)}{\phi +1+\Lambda_{n}(\frac{2\alpha }{r_{0}})-n\frac{2\alpha }{r_{0}}},$$

where *Λ*_*n*_(*x*) is the function

3.4 Λn(x)=nx−1+n∑k=0n(−1) jn+j(n+j j)(n j)x−j∑ j=0nj(−1) jn+j(n+j j)(n j)x−j,

with (n j) the binomial coefficient ‘*n* choose *j*’. Equation (3.3) holds provided *α* < *r*_0_/2 (see §3.2 for a discussion). The first term in the numerator of equation (3.3) represents the effects of the viscosity mismatch, the second term embodies the effects of surface tension, while the final term shows the effects of activity.

As *Λ*_*n*_(0) = 0 for all *n*, we obtain the following expression of σ_*n*_(*r*_0_) in the passive limit *α* → 0:

3.5 $$\sigma_{n}(r_{0}){|}_{\alpha =0}=\frac{1}{2}\left[\frac{n(\phi -1)}{\phi +1}-1-\frac{n(n^{2}-1)}{r_{0}^{3}(\phi +1)}\right]+\frac{1}{\phi +1}.$$

The first term (square brackets) of equation (3.5) is equivalent to the landmark result obtained by Paterson [58] for viscous fingering in a radial geometry (eqn. (10) in [58]), upon imposing the injection rate *Q* in Paterson’s formula equal to the total amount of the droplet’s growth per unit time in our setting (that is, $Q=\pi r_{0}^{2}$ in dimensionless variables). The last term 1/(*ϕ* + 1), however, distinguishes our result from Paterson’s, and stems from the fact that, here, the invading fluid also grows within the fingers.

**Figure 3.**
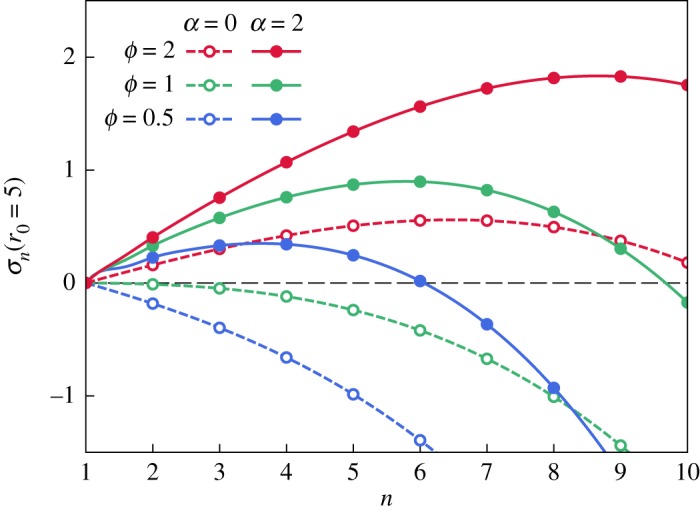
Growth rate σ_*n*_(*r*_0_ = 5) of periodic interfacial perturbations as a function of the number of fingers *n*, for a droplet of size *r*_0_ = 5, made of a passive (*α* = 0; dashed lines) or an active (*α* = 2; solid lines) fluid, with varying *ϕ*.

Figure 3 shows σ_*n*_(*r*_0_) versus *n* for various values of *ϕ* and *α*, and for an unperturbed droplet radius *r*_0_ = 5. Only integer values of *n* (circles in figure 3) have a physical interpretation. The first mode *n* = 1 corresponds to a translation of the droplet, and as *Λ*_1_(*x*) = 0 for all *x*, σ_1_(*r*_0_) = 0 for all values of *r*_0_, *α* and *ϕ*. Higher modes *n* ≥ 2 correspond to the formation of *n* fingers on the interface of the active droplet and, if unstable (that is, if σ_*n*_(*r*_0_) > 0), could potentially initiate the multicellular protrusions observed in tumours [4].

In passive fluids, *ϕ* > 1 (that is, the invaded fluid is more viscous than the invading one) is a necessary condition for fingering to be initiated, as instabilities can only grow when the pressure gradient near the interface is lower in the invading fluid [69]. However, we observe that σ_*n*_(*r*_0_) increases with *α*, so that modes that are stable when *α* = 0 may become unstable in the presence of activity *α* > 0 (compare the dashed and solid blue curves, obtained with *ϕ* = 0.5, in figure 3). Therefore, activity can trigger fingering in systems that are stable otherwise, as well as enhance and/or change the dominant modes in droplets that are already unstable.

Activity lowers the pressure gradient of the invading fluid near the interface, hence promoting instabilities. Using the expression of *p*_0_ and *p*_0_′ given in equations (2.5), we find that the condition for fingering, $\nabla p{|}_{\rho =r_{0}}<\nabla p^{\prime }{|}_{\rho =r_{0}}$, is equivalent to *ϕ* + 2*α*/*r*_0_ > 1 when the effects of surface tension are negligible.

### 3.2. High-activity regime

As already mentioned, |**v**_s_| = *α*/*β* ∼ 10^−9^ m s^−1^ is a characteristic velocity at which the active fluid would move in an unbounded space, under uniform pressure and without growth. If |**v**_s_| exceeds the growth-generated velocity at the interface, the active fluid’s motion is frustrated and further instabilities occur across its entire area. We call this regime, for which dimensionless *α* > *r*_0_/2, ‘high activity’. In this case, the derivation of equation (3.3) presented in the appendix, which assumes that perturbations are only arising at the interface and decaying away from it, is not valid. This regime is potentially relevant in the behaviour of real epithelial tissues, in which fingering at the boundaries is accompanied by velocity swirls forming across the entire area of the tissue [23].

Hence, equation (3.3) is only valid for the ‘low activity’ regime (*α* < *r*_0_/2). Yet, even in this regime, the active droplet may feature a region of instabilities near its centre *ρ* < 2*α*, where the velocity magnitude |**v**_0_| (given by equation (2.5*a*)) is less than |**v**_s_|. In particular, this situation would have also occurred in the history of the system considered in figure 3. In practice, a separate simulation-based study would be most appropriate to obtain the velocity field throughout the whole active region and to further examine the high-activity regime. In further sections of this paper, we only examine the system’s behaviour in the low-activity regime.

### 3.3. Onset of fingering

For small enough radii, surface tension stabilizes the active droplet, but its strength decreases as that droplet grows. Therefore, there exists a critical radius for the onset of fingering, below which the active droplet grows circular and unperturbed. As *n* = 2 is always the first mode to become unstable, *r*_c_(*α*, *ϕ*) defined by the conditions σ_2_(*r*_0_ = *r*_c_) = 0 and $\partial_{r_{0}}\sigma_{2}(r_{0}){|}_{r_{0}=r_{c}}>0$ provides an estimate of that critical radius. Using equation (3.3) with *Λ*_2_(*x*) = *x*(3*x* − 4)/(2*x* − 3), these conditions are equivalent to finding a polynomial root (first condition) within a subdomain (second condition), and have a unique positive solution.

**Figure 4.**
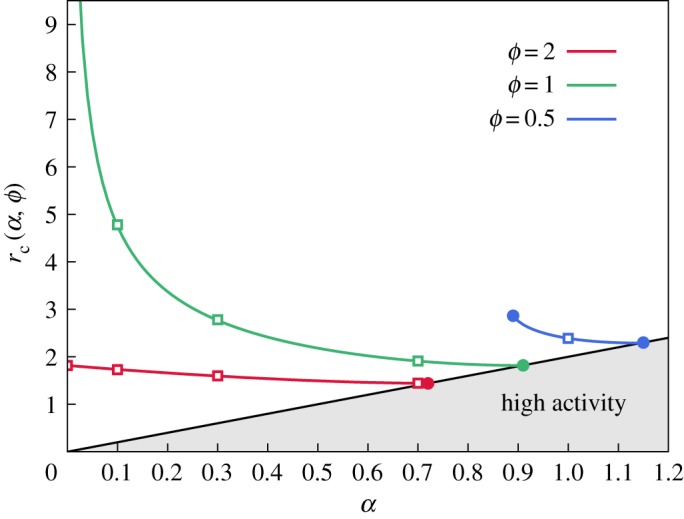
Minimum radius $r_{c}(\alpha ,\phi )$ for the initiation of fingering. The grey area on the graph signifies the high-activity regime, in which the linear stability analysis does not apply (see §3.2). The squares indicate the values of *α* used in figure 5 for each viscosity ratio *ϕ*.

We plot *r*_c_(*α*, *ϕ*) versus *α* in figure 4, which shows that increasing activity decreases the minimum radius for fingering. When *ϕ* < 1 (blue curve in figure 4), there exists a minimum value of *α*, below which fingering cannot occur, because the higher viscosity of the invading droplet has a stabilizing effect. When *ϕ* = 1 (green curve in figure 4), a moderate increase of *α* may decrease *r*_c_ multiple times. The impact of *α* in fingering is, however, reduced when *ϕ* > 1 (the red curve in figure 4), because in that case the interface would be unstable even without activity.

The range of dimensionless *r*_c_, presented in figure 4, would correspond to a radius of 10–100 µm; however, it can be much higher for lower values of *α*. This range of *r*_c_ is nevertheless in qualitative agreement with the tumour size at which the onset of fingering occurred in experimentally studied carcinomas [4].

### 3.4. Dominant mode

We now address the question of how many fingers are visible in practice, or, technically speaking, the question of which perturbation mode dominates during growth. Viscous fingering studies suggest the dominant mode is the one satisfying the so-called maximum-amplitude criterion [70]. The criterion is satisfied by the mode *n*_d_ experiencing the largest total aggregated growth in amplitude ζ_*n*_ over the entire history of the system [70]. Following [70], we obtain ζ_*n*_ by integrating the rate of the perturbation’s growth, as predicted by our linear stability analysis, over that history,

3.6 $$\zeta_{n}(r_{0})=\exp\left[\int_{R_{n}}^{r_{0}}\sigma_{n}(r)\frac{\text{d}t}{\text{d}r}\text{d}r\right],$$

where *R*_*n*_ is the radius at which mode *n* is first destabilized (i.e. the minimum radius at which σ_*n*_(*R*_*n*_) becomes positive). The dominant mode *n*_d_ is then obtained for each *r*_0_ from the conditions,

3.7*a* $$\partial_{n}\zeta_{n}(r_{0}){|}_{n=n_{d}}=0$$

and

3.7*b* $$\partial_{nn}^{2}\zeta_{n}(r_{0}){|}_{n=n_{d}}<0,$$

used to locate the maximum aggregated growth.

As we shall see, the selection of the dominant mode depends on the particular kinetics of the tumour growth. Some experimental studies have shown that an initially exponential growth [71] (corresponding to a constant *k*) subsequently slows down with time (implying a decrease in average *k*) as the tumour enlarges and its resource supply becomes a limiting factor [72–74]. Other kinetics have been measured for various tumours and phases of growth, including sigmoidal regimes in which the growth stalls [72–74]. The kinetics where the tumour’s radius grows linearly with time also naturally emerges when the tumour proliferates only within an outer rim [74]. Such growth can also occur as a temporary feature in a sigmoidal kinetics.

We thus proceed to discuss in detail the selection of dominant modes in two kinetic models of tumour growth: an exponentially growing tumour, where *k* is uniform and independent of time and $r_{0}(t)=r_{i}\;{\text{e}}^{k(t-t_{i})/2}$ (from equation (2.1*b*) at the interface, and with dimensional variables; *r*_i_ being the initial radius at time *t*_i_); and a tumour with a radius growing linearly with time, *r*_0_(*t*) = *r*_i_ + *ν*(*t* − *t*_i_), with *ν* the constant and uniform velocity of the unperturbed interface. In the latter case, the growth rate *k* appearing in equation (2.1*b*) evolves with time.

#### 3.4.1. Exponential growth

**Figure 5.**
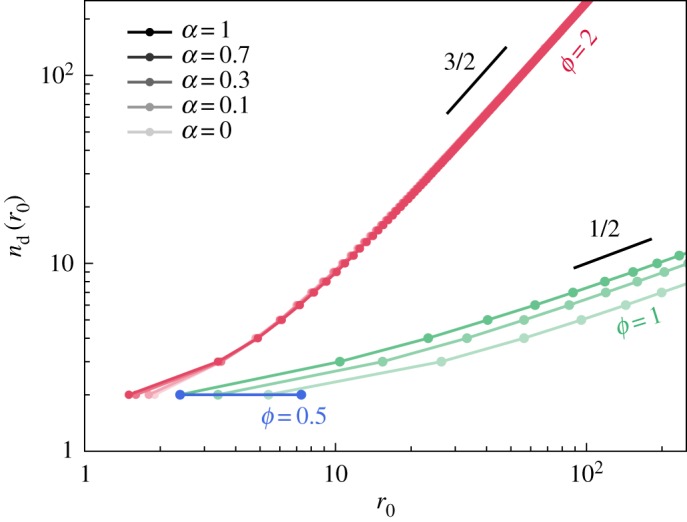
Numerical estimation of the dominant mode *n*_d_(*r*_0_) observed in an active droplet of radius *r*_0_, undergoing exponential growth, for various values of *α* and *ϕ*.

The integral given in equation (3.6) cannot be expressed analytically for all values of *ϕ* and *α*, and we plot in figure 5 the relationship *n*_d_(*r*_0_) obtained from numerical evaluation. When *ϕ* > 1, and for low radii close to the onset of fingering, we observe that the activity has only a moderate influence on the selection of the dominant mode. For later growth, when *r*_0_ becomes large, the viscosity mismatch is the governing cause of fingering and the activity plays no role in the selection of the dominant mode. We numerically observe the power-law variations $n_{d}\propto r_{0}^{3/2}$, independent of *α*. When *ϕ* = 1, higher activities promote the selection of higher modes. We obtain numerically, and for large *r*_0_, the scaling $n_{d}\propto r_{0}^{1/2}$, where the $\frac{1}{2}$ power law is independent of *α*. When *ϕ* < 1 and *α* is sufficiently high, some low-*n* modes will become destabilized. However, these perturbations will re-stabilize and decay as *r*_0_ increases further, because the stabilization from viscosity mismatch dominates as the radius of the droplet grows: active terms of equation (3.3) vanish when *r*_0_ → ∞, while terms involving *ϕ* remain constant in this limit.

We may recover analytically the observed scalings for large *n*_d_ and *r*_0_, and for *ϕ* ≥ 1. We derive in appendix B.1 the following results when *r*_0_ → ∞: $n_{d}\approx {\left(\phi -1\right)}^{1/2}\times r_{0}^{3/2}$ for *ϕ* > 1 and $n_{d}\approx {\left[{\left(\alpha /2\right)}^{2}+{\left(\alpha /2\right)}^{1/2}\right]}^{1/2}\times r_{0}^{1/2}$ for *ϕ* = 1, which we give below in dimensional variables to highlight the influence of the various physical parameters:

$$n_{d}\approx \{\begin{array}{cc} c^{1/2}{\left[\frac{k(\beta^{\prime }-\beta )}{2\gamma }\right]}^{1/2}\times r_{0}^{3/2}\;\;\;\;\;\mathrm{for}\;\beta^{\prime }\;>\beta ,\;\; & \left(3.8a\right)\\ {\left[\frac{2\gamma }{\beta k}{\left(\frac{\alpha }{4\gamma }\right)}^{2}+{\left(\frac{\alpha }{4\gamma }\right)}^{1/2}\right]}^{1/2}\times r_{0}^{1/2}\;\mathrm{for}\;\beta^{\prime }=\beta , & \left(3.8b\right) \end{array}\;$$

when *r*_0_ ≫ ${\left(\frac{2\gamma }{\beta k}\right)}^{1/3}$, and with *c* ≈ 0.06 defined as the smaller of the two solutions to 3*c* = 3 + ln*c*.

#### 3.4.2. Linear growth

We also investigate pattern selection for a tumour with a radius growing linearly with time. The derivation of σ_*n*_(*r*_0_) proceeds along identical lines, although in this case, the integral in equation (3.6) can be expressed analytically. Details of this calculation are given in appendix B.2, and in this case we find that *n*_d_ ≈ *c*(*α* + *ϕ* − 1) ^1/2^ × *r*_0_ is valid for all values of the physical parameters in the low activity regime when *r*_0_ → ∞, and where *c* ≈ 0.06 is the constant defined previously. We thus write, in dimensional form,

3.9 $$n_{d}\approx c{\left[\frac{\alpha +\nu (\beta^{\prime }-\beta )}{\gamma }\right]}^{1/2}\times r_{0},$$

when *r*_0_ ≫ ${\left(\frac{\gamma }{\beta \nu }\right)}^{1/2}$ (note that the dimensionless variables are defined differently in the linear growth, as explained in appendix B.2). The difference in the *n*_d_ versus *r*_0_ power-law dependency between equations (3.8) and (3.9) highlights the role of the growth kinetics in the fingering pattern, and is discussed with more details in the following.

#### 3.4.3. Comparison and discussion

We now examine the evolution of the tumour’s shape in the two growth kinetics studied above. An initially circular droplet is allowed to evolve, with the *n*-mode perturbation starting when the radius *r*_0_ reaches *R*_*n*_, and with an initial amplitude of 0.2 (corresponding to approx. 2 µm). The perturbation is subsequently allowed to grow according to equation (3.6), such that ζ_*n*_(*r*_0_) represents the weight of the *n*-mode at the unperturbed droplet radius *r*_0_. We further assigned a random phase difference between each *n*-mode perturbation.

**Figure 6.**
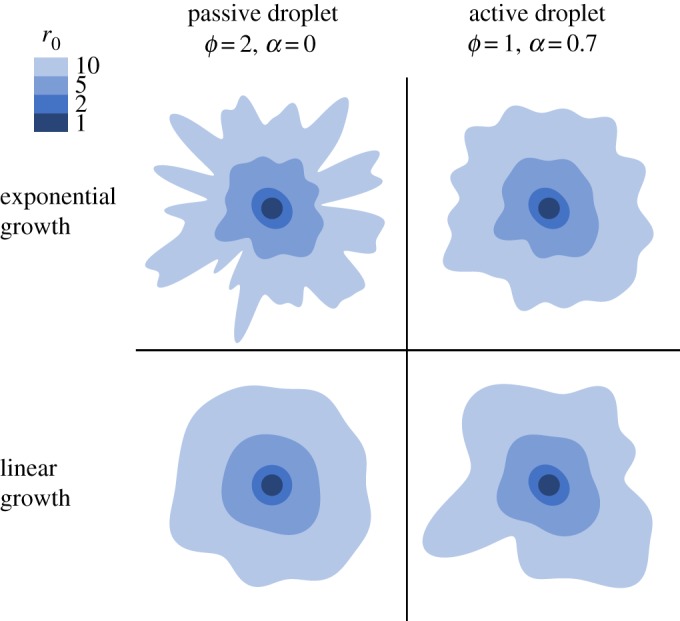
Evolution of a droplet, undergoing exponential (upper row) or linear (lower row) growth, made of a passive (left column) or active (right column) fluid. For comparison, the patterns are shown at the indicated values of the unperturbed radii *r*_0_ (see the scale of shades), even though these are reached at different times in each growth regime. We used the initial amplitude 0.2 for all modes and, to facilitate the morphological comparison, equate the characteristic lengths introduced for each growth kinetics (see *ℓ* defined in §2.4 for the exponential growth, and in appendix B.2 for the linear growth): ${\left(\frac{2\gamma }{\beta k}\right)}^{1/3}$ = ${\left(\frac{\gamma }{\beta v}\right)}^{1/2}$ ⇔ 4*βν*^3^ = *γk*^2^.

We present in figure 6 examples of droplet patterns obtained with this procedure, where fingering is driven by either viscosity mismatch (left) or by activity (right), in both the exponential (top) and linear (bottom) growth regimes. In the linear growth, activity-driven fingers emerge more distinctively than in the passive droplet; the opposite is observed in the exponential growth. These results, as well as the analytical scalings presented above, demonstrate that the role of activity in fingering depends on the kinetics of the tumour’s growth, and is indeed enhanced in the slower, linear growth kinetics. This assessment could potentially provide a basis for the mechanism behind the onset of metastasis, when the bulk growth of the primary tumour slows down or saturates.

The results presented in figures 5 and 6 relate direct observables of the tumour’s geometry, and such measurements should indeed be envisaged by experimentalists. Note, however, from figure 6 that the number of fingers (approx. 10) visible at *r*_0_ ∼ 100 µm when *ϕ* = 1 is in agreement with the experimental observations shown by Cheung *et al.* [4].

## 4. Conclusion

We have devised a model of a growing and self-propelled tissue that isolates the role of four mechanical parameters (summarized in table 1) on its dynamics. The theory is based on experimental evidence and is analytically trackable. We used it to describe the evolution of an embedded two-dimensional circular droplet that could model a carcinoma in an epithelial layer. In this example, we were able to highlight the basic mechanical conditioning required to form interfacial instabilities, reminiscent of the classical viscous fingering, and that could explain the tumour protrusions observed at the onset of metastasis. We notably find that the tissue’s active traction and growth kinetics are central to shape the instabilities’ pattern and evolution.

Our model, and the example of its application presented here, could further help predict the minimum tumour size for metastasis, as well as the number of subsequent invasive fingers emerging from the initial mass. To the best of our knowledge, these observable geometric quantities have yet to be measured systematically in experimental studies.

The relative analytical simplicity of our model allows the investigation of more complex settings, such as heterogeneous tumours where active forces and/or growth are not uniform, or processes where these parameters are evolving with time or are dependent on one another. It also offers constitutive equations that can be used in simulations, and we envisage such studies for systems with high traction forces, where active motions are faster than the growth velocity, and which may indeed be relevant in aggressive forms of cancer.

## Data accessibility

This article has no additional data.

## Appendix A. Linear stability analysis

We here follow the lines of the demonstration given by Paterson [58]. We substitute the linearized perturbations to the hydrodynamic fields in both fluids, (*δ**p*, *δ**p*′, *δ***v**, *δ***v**′), into equations (2.1)–(2.2) and obtain, to linear order

A 1*a* $$\partial_{\rho }\delta p=-\delta v_{\rho },$$

A 1*b* $$v_{\rho }\partial_{\theta }\delta p=\alpha \rho \delta v_{\theta }-\rho v_{\rho }\delta v_{\theta }$$

and

A 1*c* $$\partial_{\rho }(\rho \delta v_{\rho })=-\partial_{\theta }\delta v_{\theta },$$

in the active fluid, and

A 2*a* $$\partial_{\rho }\delta p^{\prime }=-\phi \delta v_{\rho }^{\prime },$$

A 2*b* $$\partial_{\theta }\delta p=-\rho \phi \delta v_{\theta }^{\prime }$$

and

A 2*c* $$\partial_{\rho }(\rho \delta v_{\rho }^{\prime })=-\partial_{\theta }\delta v_{\theta }^{\prime },$$

in the passive fluid.

Introducing the ansatz of periodicity in *θ*, that is (*δ**p*, *δ**p*′, *δ***v**, *δ***v**′) ∝ e^i*n**θ*^, in equations (A 1)–(A 2) allows us to calculate the partial derivatives with respect to *θ*. Combining the resulting equations, and using the expression of **v**_0_ of equation (2.5*a*), leads to second-order differential equations for the pressure perturbations in both fluids,

A 3*a* $$n^{2}\delta p=(\rho -2\alpha )\partial_{\rho }(\rho \partial_{\rho }\delta p)$$

and

A 3*b* $$n^{2}\delta p^{\prime }=\rho \partial_{\rho }(\rho \partial_{\rho }\delta p^{\prime }).$$

We now assume *α* < *r*_0_/2 (see §3.2). Equations (A 3) are both solved by linear combinations of two functions. One function in each of these combinations has incorrect asymptotic behaviour (diverging, instead of decaying away from the interface) and is removed. The retained functions serve to formulate the allowed forms of pressure perturbations,

A 4*a* δp=q(t)[∑ j=0n(−1) jn+j(n+j j)(n j)(2αρ)−j]einθ

and

A 4*b* $$\delta p^{\prime }=q^{\prime }(t){\left(\frac{\rho }{r_{0}}\right)}^{-n}{\text{e}}^{in\theta }.$$

The functions *q*(*t*) and *q*′(*t*) are then obtained by finding **δ***v*_*ρ*_ (using equation (A 4*a*) in equation (A 1*a*)) and **δ***v*′_*ρ*_ (using equation (A 4*b*) in equation (A 2*a*)), and substituting the resulting expressions into the kinematic boundary conditions, equation (2.3). One obtains the following:

A 5*a* $$\delta p=\frac{r_{0}}{n}\left[\partial_{t}\delta r-\frac{\delta r}{2}\right]\left[1+\frac{2\alpha }{r_{0}}-\Lambda_{n}\left(\frac{2\alpha }{\rho }\right)-(n-1)\frac{2\alpha }{\rho }\right]$$

and

A 5*b* $$\delta p^{\prime }=\frac{\phi r_{0}}{n}\left[\partial_{t}\delta r+\frac{\delta r}{2}\right]{\left(\frac{\rho }{r_{0}}\right)}^{-n},$$

with *Λ*_*n*_ the function defined by equation (3.4), and where the *θ*-dependency e^i*n**θ*^ has been incorporated into **δ***r*. Using both expressions in the pressure boundary condition, equation (2.4) linearized to first order in **δ***r*, and substituting **δ***r* ∝ *f*_*n*_(*t*) e^i*n**θ*^ leads to an expression for ∂_*t*_*f*_*n*_/*f*_*n*_. Upon using the definition of σ_*n*_(*r*_0_), equation (3.2), we finally obtain equation (3.3).

## Appendix B. Scaling laws for the dominant modes

**B.1. Exponential growth**

We obtain the scaling of equation (3.8*a*) for the viscosity-controlled fingering, *ϕ* > 1, by only considering the passive case (*α* = 0). The result is valid even when *α* > 0, as effects of the viscosity mismatch dominate effects of activity when *r*_0_ → ∞, as also indicated by the numerical results shown in figure 5. We then use the expression σ_*n*_(*r*_0_)|_*α*=0_ given by equation (3.5) into equation (3.6), in which we substitute d*t* = 2d*r*/*r* for exponential growth. We calculate

$$\zeta_{n}(r_{0}){|}_{\alpha =0}={\left[A_{n}\exp(A_{n}^{-1}-1)\right]}^{B_{n}},$$

with $A_{n}=\frac{\phi -1}{n(n+1)}r_{0}^{3}$ and $B_{n}=\frac{\left(n-1)(\phi -1\right)}{3(\phi +1)}$, by using the expression of *R*_*n*_ obtained from solving σ_*n*_(*R*_*n*_)|_*α*=0_ = 0. The dominant mode *n*_d_ is calculated for each *r*_0_ from the conditions of equations (3.7). Taking the limit *r*_0_ → ∞, and reintroducing dimensional variables, lead to equation (3.8*a*).

When *ϕ* = 1, the selection of the dominant mode depends on the activity *α*. We first Taylor expand *Λ*_*n*_(*x*) to first order in *x*,

B 1 $$\Lambda_{n}(x)\approx \frac{2n(n-1)}{2n-1}x.$$

This term grows linearly with *n* for *n* → ∞, while higher order coefficients in *x* plateau in this limit. Therefore, this expression of *Λ*_*n*_ is efficient even for *x* ∼ 1, and we use it to approximate $R_{n}\approx {\left[\frac{\left(n+1)(2n-1\right)}{4\alpha }\right]}^{1/2}$ and $\sigma_{n}(r_{0})\approx \frac{n(n-1)}{4r_{0}^{3}}\left[\frac{\alpha (n^{2}+n-4r_{0}^{3})}{\left(2n-1\right)r_{0}}-n-1\right]$. Upon substituting these approximations into equation (3.6), the dominant mode conditions equations (3.7) lead to equation (3.8*b*) for *r*_0_ → ∞.

**B.2. Linear growth**

In this kinetics, the growth rate *k* is not a constant, as opposed to the velocity *ν* of the unperturbed interface which is now the adequate growth parameter. We thus use a different set of dimensionless variables, based on *ν*. The characteristic length is now $\ell ={\left(\frac{\gamma }{\beta \nu }\right)}^{1/2}$, and times are re-scaled by *ℓ*/*ν*, activities by $\alpha^{*}=\beta \nu$, pressures by $p^{*}=\beta \ell \nu$ and velocities by *ν*. The derivation presented in appendix A proceeds similarly, and we find that the rate of perturbation’s growth σ_*n*_(*r*_0_) in this system of variables is written as

B 2 $$\sigma_{n}(r_{0})=\frac{1}{r_{0}}\frac{(\phi -1)(n-1)-n(n^{2}-1)/r_{0}^{2}+\Lambda_{n}(\alpha )}{\phi +1+\Lambda_{n}(\alpha )-n\alpha }\;.$$

The integral of equation (3.6), with the change of variables now written d*t* = d*r*, may then be calculated analytically and we obtain the following:

B 3 $$\zeta_{n}(r_{0})={\left[A_{n}\exp(A_{n}^{-1}-1)\right]}^{B_{n}}$$

with $A_{n}=\frac{\left(\phi \;-1)(n-1)+\Lambda_{n}(\alpha \right)}{n(n^{2}-1)}r_{0}^{2}$ and $B_{n}=\frac{1}{2}\frac{\left(\phi -1)(n-1)+\Lambda_{n}(\alpha \right)}{\phi +1+\Lambda_{n}(\alpha )-n\alpha }$. Upon using the approximation of *Λ*_*n*_(*x*) given by equation (B 1), the conditions equations (3.7) now imply equation (3.9) in the limit *r*_0_ → ∞.

## References

[1] Tracqui P 2009 Biophysical models of tumour growth. Rep. Prog. Phys. 72, 056701 (10.1088/0034-4885/72/5/056701)

[2] Friedl P, Gilmour D 2009 Collective cell migration in morphogenesis, regeneration and cancer. Nat. Rev. Mol. Cell Biol. 10, 445– 457. (10.1038/nrm2720) 19546857

[3] Friedl P, Wolf K 2003 Tumour-cell invasion and migration: diversity and escape mechanisms. Nat. Rev. Cancer 3, 362–374. (10.1038/nrc1075) 12724734

[4] Cheung KJ, Gabrielson E, Werb Z, Ewald AJ 2013 Collective invasion in breast cancer requires a conserved basal epithelial program. Cell 155, 1639–1651. (10.1016/j.cell.2013.11.029) 24332913 PMC3941206

[5] Wong IY, Javaid S, Wong EA, Perk S, Haber DA, Toner M, Irimia D 2014 Collective and individual migration following the epithelial–mesenchymal transition. Nat. Mater. 13, 1063–1071. (10.1038/nmat4062) 25129619 PMC4209198

[6] Westcott JM, Prechtl AM, Maine EA, Dang TT, Esparza MA, Sun H, Zhou Y, Xie Y, Pearson GW 2015 An epigenetically distinct breast cancer cell subpopulation promotes collective invasion. J. Clin. Invest. 125, 1927–1943. (10.1172/JCI77767) 25844900 PMC4463195

[7] Carey SP, Starchenko A, McGregor AL, Reinhart-King CA 2013 Leading malignant cells initiate collective epithelial cell invasion in a three-dimensional heterotypic tumor spheroid model. Clin. Exp. Metastasis 30, 615–630. (10.1007/s10585-013-9565-x) 23328900 PMC3646083

[8] Haeger A, Wolf K, Zegers MM, Friedl P 2015 Collective cell migration: guidance principles and hierarchies. Trends Cell Biol. 25, 556–566. (10.1016/j.tcb.2015.06.003) 26137890

[9] Sporn MB 1996 The war on cancer. The Lancet 347, 1377–1381. (10.1016/S0140-6736(96)91015-6) 8637346

[10] Petitjean L, Reffay M, Grasland-Mongrain E, Poujade M, Ladoux B, Buguin A, Silberzan P 2010 Velocity fields in a collectively migrating epithelium. Biophys. J. 98, 1790–1800. (10.1016/j.bpj.2010.01.030) 20441742 PMC2862185

[11] Klarlund JK 2012 Dual modes of motility at the leading edge of migrating epithelial cell sheets. Proc. Natl Acad. Sci. USA 109, 15 799–15 804. (10.1073/pnas.1210992109) 23019364 PMC3465438

[12] Khain E, Sander LM 2006 Dynamics and pattern formation in invasive tumor growth. Phys. Rev. Lett. 96, 188103 (10.1103/PhysRevLett.96.188103) 16712401

[13] Bizzarri M, Cucina A, Conti F, D’Anselmi F 2008 Beyond the oncogene paradigm: understanding complexity in cancerogenesis. Acta Biotheor. 56, 173–196. (10.1007/s10441-008-9047-8) 18288572

[14] Basan M, Joanny JF, Prost J, Risler T 2011 Undulation instability of epithelial tissues. Phys. Rev. Lett. 106, 158101 (10.1103/PhysRevLett.106.158101) 21568616

[15] Ciarletta P 2013 Buckling instability in growing tumor spheroids. Phys. Rev. Lett. 110, 158102 (10.1103/PhysRevLett.110.158102) 25167314

[16] Goriely A, Ben Amar M 2005 Differential growth and instability in elastic shells. Phys. Rev. Lett. 94, 198103 (10.1103/PhysRevLett.94.198103) 16090217

[17] Ben Amar M, Ciarletta P 2010 Swelling instability of surface-attached gels as a model of soft tissue growth under geometric constraints. J. Mech. Phys. Solids 58, 935–954. (10.1016/j.jmps.2010.05.002)

[18] Basan M, Elgeti J, Hannezo E, Rappel WJ, Levine H 2013 Alignment of cellular motility forces with tissue flow as a mechanism for efficient wound healing. Proc. Natl Acad. Sci. USA 110, 2452–2459. (10.1073/pnas.1219937110) 23345440 PMC3574962

[19] Köpf MH, Pismen LM 2013 A continuum model of epithelial spreading. Soft Matter 9, 3727–3734. (10.1039/c3sm26955h)

[20] Ouaknin GY, Bar-Yoseph PZ 2009 Stochastic collective movement of cells and fingering morphology: no maverick cells. Biophys. J. 97, 1811–1821. (10.1016/j.bpj.2009.05.064) 19804711 PMC2756401

[21] Ferreira SC, Martins ML, Vilela MJ 2002 Reaction-diffusion model for the growth of avascular tumor. Phys. Rev. E 65, 021907 (10.1103/PhysRevE.65.021907) 11863563

[22] Guiot C, Pugno N, Delsanto PP 2006 Elastomechanical model of tumor invasion. Appl. Phys. Lett. 89, 233901 (10.1063/1.2398910)

[23] Poujade M, Grasland-Mongrain E, Hertzog A, Jouanneau J, Chavrier P, Ladoux B, Buguin A, Silberzan P 2007 Collective migration of an epithelial monolayer in response to a model wound. Proc. Natl Acad. Sci. USA 104, 15 988–15 993. (10.1073/pnas.0705062104) 17905871 PMC2042149

[24] Guiot C, Delsanto PP, Deisboeck TS 2007 Morphological instability and cancer invasion: a ‘splashing water drop’ analogy. Theor. Biol. Med. Modell. 4, 4 (10.1186/1742-4682-4-4) PMC1794228 17254360

[25] Hallou A, Jennings J, Kabla AJ 2017 Tumour heterogeneity promotes collective invasion and cancer metastatic dissemination. R. Soc. open sci. 4, 161007 (10.1098/rsos.161007) 28878958 PMC5579073

[26] Gordon VD, Valentine MT, Gardel ML, Andor-Ardó D, Dennison S, Bogdanov AA, Weitz DA, Deisboeck TS 2003 Measuring the mechanical stress induced by an expanding multicellular tumor system: a case study. Exp. Cell Res. 289, 58–66. (10.1016/S0014-4827(03)00256-8) 12941604

[27] du Roure O, Saez A, Buguin A, Austin RH, Chavrier P, Silberzan P, Ladoux B 2005 Force mapping in epithelial cell migration. Proc. Natl Acad. Sci. USA 102, 2390–2395. (10.1073/pnas.0408482102) 15695588 PMC548966

[28] Mierke CT, Rösel D, Fabry B, Brábek J 2008 Contractile forces in tumor cell migration. Eur. J. Cell Biol. 87, 669–676. (10.1016/j.ejcb.2008.01.002) 18295931 PMC2566782

[29] Trepat X, Wasserman MR, Angelini TE, Millet E, Weitz DA, Butler JP, Fredberg JJ 2009 Physical forces during collective cell migration. Nat. Phys. 5, 426–430. (10.1038/nphys1269)

[30] Blanch-Mercader C, Vincent R, Bazellières E, Serra-Picamal X, Trepat X, Casademunt J 2017 Effective viscosity and dynamics of spreading epithelia: a solvable model. Soft Matter 13, 1235–1243. (10.1039/C6SM02188C) 28098306

[31] Mark S, Shlomovitz R, Gov NS, Poujade M, Grasland-Mongrain E, Silberzan P 2010 Physical model of the dynamic instability in an expanding cell culture. Biophys. J. 98, 361–370. (10.1016/j.bpj.2009.10.022) 20141748 PMC2814206

[32] Nagilla A, Prabhakar R, Jadhav S 2018 Linear stability of an active fluid interface. Phys. Fluids 30, 022109 (10.1063/1.5012109)

[33] Zimmermann J, Basan M, Levine H 2014 An instability at the edge of a tissue of collectively migrating cells can lead to finger formation during wound healing. Eur. Phys. J. Spec. Top. 223, 1259–1264. (10.1140/epjst/e2014-02189-7)

[34] Nesbitt D, Pruessner G, Lee CF 2017 Edge instability in incompressible planar active fluids. Phys. Rev. E 96, 062615 (10.1103/PhysRevE.96.062615) 29347377

[35] Saffman PG, Taylor G 1958 The penetration of a fluid into a porous medium or Hele-Shaw cell containing a more viscous liquid. Proc. R. Soc. Lond. A 245, 312–329. (10.1098/rspa.1958.0085)

[36] Cristini V, Lowengrub J, Nie Q 2003 Nonlinear simulation of tumor growth. J. Math. Biol. 46, 191–224. (10.1007/s00285-002-0174-6) 12728333

[37] Arciero JC, Mi Q, Branca MF, Hackam DJ, Swigon D 2011 Continuum model of collective cell migration in wound healing and colony expansion. Biophys. J. 100, 535–543. (10.1016/j.bpj.2010.11.083) 21281567 PMC3030184

[38] Ravasio A *et al.* 2015 Gap geometry dictates epithelial closure efficiency. Nat. Commun. 6, 7683 (10.1038/ncomms8683) 26158873 PMC4510701

[39] Friedl P, Wolf K 2010 Plasticity of cell migration: a multiscale tuning model. J. Cell Biol. 188, 11–19. (10.1083/jcb.200909003) 19951899 PMC2812848

[40] Lambert M, Thoumine O, Brevier J, Choquet D, Riveline D, Mège RM 2007 Nucleation and growth of cadherin adhesions. Exp. Cell Res. 313, 4025–4040. (10.1016/j.yexcr.2007.07.035) 17765222

[41] Jennings JN 2014 A new computational model for multi-cellular biological systems. PhD thesis, University of Cambridge.

[42] Diepenbruck M, Christofori G 2016 Epithelial-mesenchymal transition (EMT) and metastasis: yes, no, maybe? Curr. Opin. Cell Biol. 43, 7–13. (10.1016/j.ceb.2016.06.002) 27371787

[43] Ranft J, Basan M, Elgeti J, Joanny JF, Prost J, Julicher F 2010 Fluidization of tissues by cell division and apoptosis. Proc. Natl Acad. Sci. USA 107, 20 863–20 868. (10.1073/pnas.1011086107) PMC3000289 21078958

[44] Blanch-Mercader C, Casademunt J 2017 Hydrodynamic instabilities, waves and turbulence in spreading epithelia. Soft Matter 13, 6913–6928. (10.1039/C7SM01128H) 28825077

[45] Szabó A, Ünnep R, Méhes E, Twal WO, Argraves WS, Cao Y, Czirók A 2010 Collective cell motion in endothelial monolayers. Phys. Biol. 7, 046007 (10.1088/1478-3975/7/4/046007) 21076204 PMC3044241

[46] Wensink HH, Dunkel J, Heidenreich S, Drescher K, Goldstein RE, Lowen H, Yeomans JM 2012 Meso-scale turbulence in living fluids. Proc. Natl Acad. Sci. USA 109, 14 308–14 313. (10.1073/pnas.1202032109) PMC3437854 22908244

[47] Selmeczi D, Mosler S, Hagedorn PH, Larsen NB, Flyvbjerg H 2005 Cell motility as persistent random motion: theories from experiments. Biophys. J. 89, 912–931. (10.1529/biophysj.105.061150) 15951372 PMC1366641

[48] Farooqui R, Fenteany G 2004 Multiple rows of cells behind an epithelial wound edge extend cryptic lamellipodia to collectively drive cell-sheet movement. J. Cell Sci. 118, 51–63. (10.1242/jcs.01577) 15585576

[49] Zaritsky A, Kaplan D, Hecht I, Natan S, Wolf L, Gov NS, Ben-Jacob E, Tsarfaty I 2014 Propagating waves of directionality and coordination orchestrate collective cell migration. PLoS Comput. Biol. 10, e1003747 (10.1371/journal.pcbi.1003747) 25058592 PMC4109844

[50] Zaritsky A, Welf ES, Tseng YY, Angeles Rabadán M, Serra-Picamal X, Trepat X, Danuser G 2015 Seeds of locally aligned motion and stress coordinate a collective cell migration. Biophys. J. 109, 2492–2500. (10.1016/j.bpj.2015.11.001) 26682808 PMC4699880

[51] Notbohm J *et al.* 2016 Cellular contraction and polarization drive collective cellular motion. Biophys. J. 110, 2729–2738. (10.1016/j.bpj.2016.05.019) 27332131 PMC4919426

[52] Banerjee S, Utuje KJC, Marchetti MC 2015 Propagating stress waves during epithelial expansion. Phys. Rev. Lett. 114, 228101 (10.1103/PhysRevLett.114.228101) 26196647

[53] Méhes E, Vicsek T 2014 Collective motion of cells: from experiments to models. Integr. Biol. 6, 831–854. (10.1039/C4IB00115J) 25056221

[54] Bi D, Yang X, Marchetti MC, Manning ML 2016 Motility-driven glass and jamming transitions in biological tissues. Phys. Rev. X 6, 021011 (10.1103/physrevx.6.021011) 28966874 PMC5619672

[55] Toner J, Tu Y 1995 Long-range order in a two-dimensional dynamical XY model: how birds fly together. Phys. Rev. Lett. 75, 4326–4329. (10.1103/PhysRevLett.75.4326) 10059876

[56] Toner J, Tu Y 1998 Flocks, herds, and schools: a quantitative theory of flocking. Phys. Rev. E 58, 4828–4858. (10.1103/PhysRevE.58.4828)

[57] Yang X, Marchetti MC 2015 Hydrodynamics of turning flocks. Phys. Rev. Lett. 115, 258101 (10.1103/PhysRevLett.115.258101) 26722945

[58] Paterson L 1981 Radial fingering in a Hele Shaw cell. J. Fluid Mech. 113, 513–529. (10.1017/S0022112081003613)

[59] Pompe T, Kaufmann M, Kasimir M, Johne S, Glorius S, Renner L, Bobeth M, Pompe W, Werner C 2011 Friction-controlled traction force in cell adhesion. Biophys. J. 101, 1863–1870. (10.1016/j.bpj.2011.08.027) 22004739 PMC3192957

[60] Ananthakrishnan R, Ehrlicher A 2007 The forces behind cell movement. Int. J. Biol. Sci. 3, 303–317. (10.7150/ijbs.3.303) 17589565 PMC1893118

[61] Manning ML, Foty RA, Steinberg MS, Schoetz EM 2010 Coaction of intercellular adhesion and cortical tension specifies tissue surface tension. Proc. Natl Acad. Sci. USA 107, 12 517–12 522. (10.1073/pnas.1003743107) PMC2906578 20616053

[62] Lecuit T, Lenne PF 2007 Cell surface mechanics and the control of cell shape, tissue patterns and morphogenesis. Nat. Rev. Mol. Cell Biol. 8, 633–644. (10.1038/nrm2222) 17643125

[63] Foty RA, Forgacs G, Pfleger CM, Steinberg MS 1994 Liquid properties of embryonic tissues: measurement of interfacial tensions. Phys. Rev. Lett. 72, 2298–2301. (10.1103/PhysRevLett.72.2298) 10055839

[64] Marchetti MC, Fily Y, Henkes S, Patch A, Yllanes D 2016 Minimal model of active colloids highlights the role of mechanical interactions in controlling the emergent behavior of active matter. Curr. Opin. Colloid Interface Sci. 21, 34–43. (10.1016/j.cocis.2016.01.003)

[65] Callan-Jones AC, Joanny JF, Prost J 2008 Viscous-fingering-like instability of cell fragments. Phys. Rev. Lett. 100, 258106 (10.1103/PhysRevLett.100.258106) 18643710

[66] Kim JH *et al.* 2013 Propulsion and navigation within the advancing monolayer sheet. Nat. Mater. 12, 856–863. (10.1038/nmat3689) 23793160 PMC3750079

[67] do Carmo MP 1976 Differential geometry of curves and surfaces. Englewood Cliffs, NJ: Prentice-Hall.

[68] Tarle V, Ravasio A, Hakim V, Gov NS 2015 Modeling the finger instability in an expanding cell monolayer. Integr. Biol. 7, 1218–1227. (10.1039/C5IB00092K) 26099063

[69] Flament C, Pacitto G, Bacri JC, Drikis I, Cebers A 1998 Viscous fingering in a magnetic fluid. I. Radial Hele-Shaw flow. Phys. Fluids 10, 2464–2472. (10.1063/1.869765)

[70] Dias EO, Miranda JA 2013 Wavelength selection in Hele-Shaw flows: a maximum-amplitude criterion. Phys. Rev. E 88, 013016 (10.1103/PhysRevE.88.013016) 23944558

[71] Withers HR, Lee SP 2006 Modeling growth kinetics and statistical distribution of oligometastases. Semin. Radiat. Oncol. 16, 111–119. (10.1016/j.semradonc.2005.12.006) 16564446

[72] Herman AB, Savage VM, West GB 2011 A quantitative theory of solid tumor growth, metabolic rate and vascularization. PLoS ONE 6, e22973 (10.1371/journal.pone.0022973) 21980335 PMC3182997

[73] Guiot C, Degiorgis PG, Delsanto PP, Gabriele P, Deisboeck TS 2003 Does tumor growth follow a ‘universal law’? J. Theor. Biol. 225, 147–151. (10.1016/S0022-5193(03)00221-2) 14575649

[74] Benzekry S, Lamont C, Beheshti A, Tracz A, Ebos JML, Hlatky L, Hahnfeldt P 2014 Classical mathematical models for description and prediction of experimental tumor growth. PLoS Comput. Biol. 10, e1003800 (10.1371/journal.pcbi.1003800) 25167199 PMC4148196

